# Comparative evaluation of long-term preservation methods for morphologically distinct bacteriophages

**DOI:** 10.1128/spectrum.01442-24

**Published:** 2025-04-16

**Authors:** Wanqi Huang, Mohammadali Khan Mirzaei, Li Deng

**Affiliations:** 1 Technical University of Munich, TUM School of Life Sciences, Central Institute of Infection Prevention (ZIP), Freising, Germany; 2 Institute of Virology, Helmholtz Centre Munich - German Research Centre for Environmental Health, Neuherberg, Germany; American Type Culture Collection, Manassas, Virginia, USA

**Keywords:** bacteriophages, biopreservation, bacteriophage therapy, phage preservation

## Abstract

The rapid increase in antibiotic resistance has led to a renewed interest in phage therapy, which has created a need for the establishment of phage collections in order to preserve diverse phages and to reduce the delivery time to patients. However, there are currently no standard methods for the long-term preservation of phages. We assessed the stability of four different phages under distinct storage conditions, including different temperatures, storage solutions, concentrations, and with or without cryoprotectant. We found that the type of storage buffer has a significant impact on phage stability, followed by the storage temperature. Phages demonstrated higher viability in lysogeny broth (LB) than saline–magnesium (SM) buffer without gelatin. We also observed a higher sensitivity to freezing in tailed phages with longer tails, such as T4. Ultimately, we found that all four phages maintained high stability after snap freezing, followed by storage at −80°C using LB as a storage buffer without cryoprotectant.

**IMPORTANCE:**

Phage therapy, which involves treating bacterial infections using bacteriophages (phage), has shown promise as an alternative to antibiotics and can offer a solution for treating infections caused by antibiotic-resistant bacteria. However, phages are not conventional drugs and can lose their viability when stored under unsuitable conditions. Their high diversity makes finding a standard storage method for long-term preservation challenging. Here, we studied the stability of phages under different storage conditions and identified key factors affecting their viability. We have also identified a specific storage condition that can effectively preserve a wide range of phage morphotypes for over 2 years.

## INTRODUCTION

The rapidly growing worldwide antimicrobial resistance crisis has renewed interest in phage therapy, and increasing clinical application of phages has been reported recently (1). Besides, phages have been applied as biocontrol agents, animal husbandry, veterinary medicine, and agriculture (2). The usage of phage products in food has been approved by the Food and Drug Administration (1).

Building a phage collection is important for the timely delivery of phage therapy to patients. This involves systematically isolating, characterizing, and storing phages. However, due to their high diversity, phages may exhibit variations in stability under storage conditions (3). Therefore, it is essential to find optimal conditions for storing phages. Phages are usually stored as broth lysates at refrigerated temperatures or sub-zero temperatures with stabilizers, like glycerol, or in liquid nitrogen for the long term. Yet, previous studies have suggested that some phages can remain stable for several years at 4°C, and most tailed phages are stable at 4°C and −80°C for years (4). Chemical-based buffers, like saline and SM buffer, are recommended and widely used for storing phages (3–7). In addition, snap freezing in liquid nitrogen has been shown to improve phage stability (8). Yet, the high diversity of phage characteristics makes it challenging to suggest a universal preservation method. Previous studies have mainly assessed individual storage conditions, using a limited number of phages, which overlooked the potential interactive effects of these conditions (4, 5, 9, 10).

Here, we systematically examine phages with varied morphologies and genomic content under different storage conditions. In addition, we assess the impact of key factors thought to significantly influence phage stability, such as initial concentrations, temperature, storage solutions, and cryoprotectants, on these phages. Phage concentrations were monitored at nine different time points over 2 years, using four distinct model coliphages (T4, T7, PhiX174, and MS2) with well-characterized properties. These phages differ in morphology and genomic composition, making them highly representative of a wide range of phages (8, 11–13).

Our results highlight the importance of storage conditions in the long-term preservation of phages. While it is difficult to choose a single method that can preserve all phages without any loss of titer, we found that snap freezing followed by storing at −80°C in LB medium without glycerol is effective in minimizing long-term titer reduction across most phages.

## MATERIALS AND METHODS

### Bacterial strains and bacteriophages

All bacterial strains and phages used in the study were purchased from the American Type Culture Collection (ATCC) and the German Collection of Microorganisms and Cell Cultures (DSMZ). *Escherichia coli* strains DSM 13127 and DSM 5695 were used as hosts of phages PhiX174 and MS2, respectively. *E. coli* ATCC 11303 was used to propagate the phages T4 and T7. Detailed information about the phages and their hosts is listed in Table 1. All strains were routinely cultivated in Luria–Bertani medium (LB, Carl Roth, Karlsruhe, Germany) at 37°C with shaking at 200rpm or on LB plates containing 1.5% (w/v) agar–agar (Carl Roth, Karlsruhe, Germany).

**TABLE 1:** Information on each phage used for the study (8, 11–15)

Phage name	Taxonomy (genus, family)	Morphotype	Structure	Phage particle size (Ø nm)	Genome type	Genome size (bp)	Bacterial host strain
T4	*Tequatrovirus, Tevenvirinae*	Myovirus	Non-enveloped, long contractile tail with sheath and fibers	100	Linear dsDNA	168,903	ATCC 11303
T7	*Teseptimavirus, Studiervirinae*	Podovirus	Non-enveloped, short non-contractile tail with fibers	58	Linear dsDNA	39,937	ATCC 11303
PhiX174	*Sinsheimervirus, Microviridae*	Microvirus	Non-enveloped, untailed,	26	Circular ssDNA	5,386	DSM 5695
MS2	*Emesvirus, Fiersviridae*	Levivirus	Non-enveloped, untailed	30	Linear ssRNA	3,569	ATCC 13127

### Phage propagation and plaque assay

LB broth was inoculated with 2% (v/v) bacterial overnight culture and incubated at 37°C for 2 h until the exponential phase. Then, 1% (v/v) phage was added into the bacterial culture, and the mixture was left to incubate for 4–5 h. After the incubation period, bacterial cells were removed by spinning at 4,000 × *g* for 20 min, followed by filtration of the supernatant through a 0.22 µm polyether sulfone syringe filter (Millex Merck Millipore, Burlington, USA).

The phage titer was determined using the double-layer plaque assay as previously described (16). Briefly, phage lysate was diluted from log (0) to log (−9), and then 100 µL of diluted phage lysate was mixed with 100 µL of bacterial overnight culture (OD_600_ 0.4, ~10^9^ CFU/mL) in 3 mL of molten soft agar (0.6% LB agar) and poured on LB plates. The plates were incubated at 37°C overnight, and the plaques were counted to estimate the phage concentration expressed as plaque-forming units per mL (PFU/mL).

### Phage buffer exchange

Before freezing the samples, the media in the phage lysates were substituted with different storage solutions to assess their impact on the stability of phages. In brief, the phage lysates were concentrated by centrifugation at 35,000 × *g* at 4°C for 3 h, and the pellets were resuspended in the following storage solutions: lysogeny broth (LB), saline–magnesium buffer (SM), SM buffer containing 0.01% gelatin (SMG), and a phosphate-based phage buffer. Then, phage suspensions were diluted to 10^8^ and 10^9^ PFU/mL with the proper buffers to assess the effect of initial concentration on phage stability. The initial titer of each phage was re-measured after dilution by plaque assay 1 day before storage. The detailed components and characteristics of the storage solutions are summarized in Table 2.

**TABLE 2:** Composition of storage solutions used in the study

Storage solution	Composition	pH	Supplier
LB (premix)	Tryptone 10 g/L Yeast extract 5 g/L Sodium chloride 10 g/L	7	Carl Roth, Karlsruhe, Germany
SM	Sodium chloride 5.8 g/L Magnesium sulfate heptahydrate 2 g/L TRIS-hydrochloride 6 g/L	7.5	Merck KGaA, Darmstadt, Germany Sigma-Aldrich, Saint Louis, USA Promega, Madison, USA
SMG	Sodium chloride 5.8 g/L Magnesium sulfate heptahydrate 2 g/L TRIS-hydrochloride 6 g/L Gelatin 0.01% (w/v)	7.5	Merck KGaA, Darmstadt, Germany Sigma-Aldrich, Saint Louis, USA Promega, Madison, USA Carl Roth, Karlsruhe, Germany
Phage buffer	Disodium phosphate anhydrous 7 g/L Potassium phosphate anhydrous 3 g/L Sodium chloride 5 g/L Magnesium sulfate 0.12 g/L Calcium chloride 0.11 g/L	7	Carl Roth, Karlsruhe, Germany Sigma-Aldrich, Saint Louis, USA ThermoFisher Scientific, Waltham, USA Avantor Performance Materials, Radnor, USA

### Phage storage at different temperatures and flash freezing in LN_2_

A total of 400 µL of phage lysates in LB medium, SM buffer, SM buffer containing 0.01% gelatin, and phage buffer was aliquoted in 0.5 mL microtubes. These lysates were stored at 4°C or frozen with 20% glycerol as a stabilizer at −20°C and −80°C. To assess the effectiveness of glycerol as a stabilizer during and after flash freezing, phage lysates were deep frozen with or without 20% glycerol in liquid nitrogen (LN_2_) for 10 min and stored at −80°C. The activity of phages stored in LB medium and SM buffer was determined after 1 week, and 1, 3, 6, 9, 12, 16, 18, and 24 months. Phage lysates in SMG and phage buffer were tested after 1, 3, 6, 9, 12, 18, and 26 months. The cryopreserved samples were thawed at room temperature.

### Lyophilization

Ten replicates of T4 and MS2 phage lysates were buffer-exchanged and diluted to the final titer of 10^10^ PFU/mL in SM buffer containing 0.5 M sucrose as a stabilizer. The lyophilization was performed as previously described (17). In brief, 1mL of each sample was frozen at −80°C in a 4 mL glass vial (Supelco, Bellefonte, USA) for 30 min. Then, lysates were lyophilized using Alpha 2-4 LDplus freeze-dryer (Martin Christ, Osterode, Germany) at −60°C and 0.010 mbar for 24 h. Once completed, the vials were screwed by a plastic top with silica gel and stored at 4°C. The freeze–dried samples were rehydrated in 1 mL SM buffer, and the phage titer was determined by plaque assay at 1 day, and 1, 6, 12, 18, and 24 months post-lyophilization.

### Phage DNA and RNA extraction

A volume of 200 µL of phage lysates was mixed with 2 µL (two units) of DNase I (1 U/µL) from ThermoFisher, Waltham, USA, and incubated at 37°C for 30 min. The DNase was then inactivated by incubation at 65°C for 50 min. Subsequently, 40 µL of 20 mg/mL Proteinase K (Carl Roth, Karlsruhe, Germany) was added to the lysates and incubated at 37°C for 30 min. Phage DNA and RNA were then extracted using the Wizard Plus Minipreps DNA purification kit (Promega, Madison, USA). The lysates were mixed with double the volume of Wizard DNA purification resin and passed slowly through the Wizard Minicolumn (Promega, Madison, USA). The column was then washed with 2 mL of 80% isopropanol, and the residual liquid was removed by centrifugation at 10,000 × *g* for 2 min. DNA/RNA was eluted in 70 µL of TE buffer preheated to 80°C after centrifugation at 10,000 × *g* for 30 s. Finally, the DNA/RNA samples were stored at −80°C until they were quantified by quantitative PCR (qPCR).

### Reverse transcription

The RNA of MS2 was first reverse transcribed to cDNA using Maxima First Strand cDNA Synthesis Kit for RT-qPCR (ThermoFisher Scientific, Waltham, USA). To do this, 1 µL of the extracted MS2 RNA was mixed with 4 µL of 5× Reaction Mix, 2 µL of Maxima Enzyme Mix, and 17 µL of nuclease-free water. The mixture was incubated at 25°C for 10 min, followed by 15 min at 50°C, and terminated at 85°C for 5 min. Finally, the cDNA was stored at −80°C before the performance of qPCR.

### Construction of qPCR standards

The qPCR standards were synthesized by amplifying the target genes of each phage using PCR with GoTaq Green Master Mix (Promega, Madison, USA). The PCR cycling and primers (Table 3) used for the four phages were adapted from previous studies (18–21). For MS2, the program started with an initial denaturation of 10 min at 95°C, followed by 40 cycles of 15 s at 95°C, 30 s at 50°C, and 30 s at 72°C (18). The cycling for Phix174 was 3 min at 94°C, followed by 40 cycles of 15 s at 94°C and 60 s at 60°C (19). For T4, the qPCR program started with 10 min at 95°C, followed by 50 cycles of 15 s at 95°C, 60 s at 60°C, and 60 s at 72°C (20). The PCR products were visualized in a 2.5% agarose gel (Biozyme, Oldendorf, Germany) and purified using a GeneJET Gel Extraction Kit (ThermoFischer Scientific, Waltham, USA). The synthesized gene fragments were quantified by Qubit dsDNA HD Assay Kit (Thermo Scientific, Waltham, USA), and the copy numbers were calculated by the following formula (22):


$$Number\;of\;copies/\mu L=\frac{6.02\times 10^{23}\left(\frac{molecules}{mole}\right)\times DNA\;concentration\;\left(\frac{g}{\mu L}\right)}{DNA\;length\;\left(bp\right)\times 660\;\left(\frac{g}{mol/bp}\right)}$$

**TABLE 3:** Oligonucleotide primer sets for qPCR

Phage (reference)	Primer sequence	Product size (bp)
MS2 (18)	MS2_2717F: 5′-CTGGGCAATAGTCAAA-3′ MS2_ 3031R: 5′-CGTGGATCTGACATAC-3′	314
PhiX174 (19)	PhiXF: 5’- ACAAAGTTTGGATTGCTACTGACC-3′ PhiXR: 5′-CGGCAGCAATAAACTCAACAGG-3′	123
T4 (20)	T4F: 5′-AAGCGAAAGAAGTCGGTGAA-3′ T4R: 5′-CGCTGTCATAGCAGCTTCAG-3′	163
T7 (21)	T7_4453F: 5′-CTGTGTCAATGTTCAACCCG-3′ T7_5008R: 5‘-GTGCCCAGCTTGACTTTCTC-3′	555

### qPCR using SYBR Green I

The quantity of DNA or RNA in each phage stock was quantified by qPCR using LightCycler 480 Real-Time PCR System (Roche, Basel, Switzerland). The process involved using 10 µL of Brilliant III Ultra-Fast SYBR Green qPCR Master Mix (Agilent Technologies, Santa Clara, USA), 2µL of template DNA or cDNA, 2µL of primer pairs, and 6µL of DNase/RNase-free water. The qPCR program used for all phages included an initial denaturation at 95°C for 5 min, followed by 45 cycles of 10 s denaturation at 95°C, 10 s annealing at 60°C, and 10 s elongation at 72°C. The specificity of the qPCR results was confirmed by melt curve analysis, which was generated by a gradual increase of temperature from 65°C to 97°C with a ramp rate of 0.11 °C/s.

### Statistics

Phage titer reductions are shown as the mean ± standard deviation based on three replicates of plaque assay for each experiment. To compare the impact of different factors on phage stability, the relative changes in phage concentration were calculated by dividing the phage concentration at month 24 by the initial phage concentrations. Statistical comparisons were conducted using the Kruskal–Wallis test, followed by Dunn’s test for pairwise comparisons, using R and GraphPad Prism, version 10.0. *P-*values less than 0.05 were considered statistically significant.

## RESULTS

We explored the impact of different storage factors on the stability of four lytic phages: T7, T4, PhiX174, and MS2. To this end, we evaluated the effect of using two commonly used storage solutions, LB broth and SM buffer, on the stability of these phages at two different initial concentrations, 10^8^ and 10^9^ PFU/mL (Table S1). In addition, we tested the effect of a phosphate-based phage buffer and SM buffer supplemented with 0.01% gelatin on PhiX174 and T4. The phage preparations were stored at temperatures of 4°C, −20°C, and −80°C with or without glycerol to study the effect of cryoprotectants on phage stability. The concentrations were determined after 1 week, and 1, 3, 6, 9, 12, 16, 18, and 24 months using plaque assay and qPCR.

**Fig 1:**
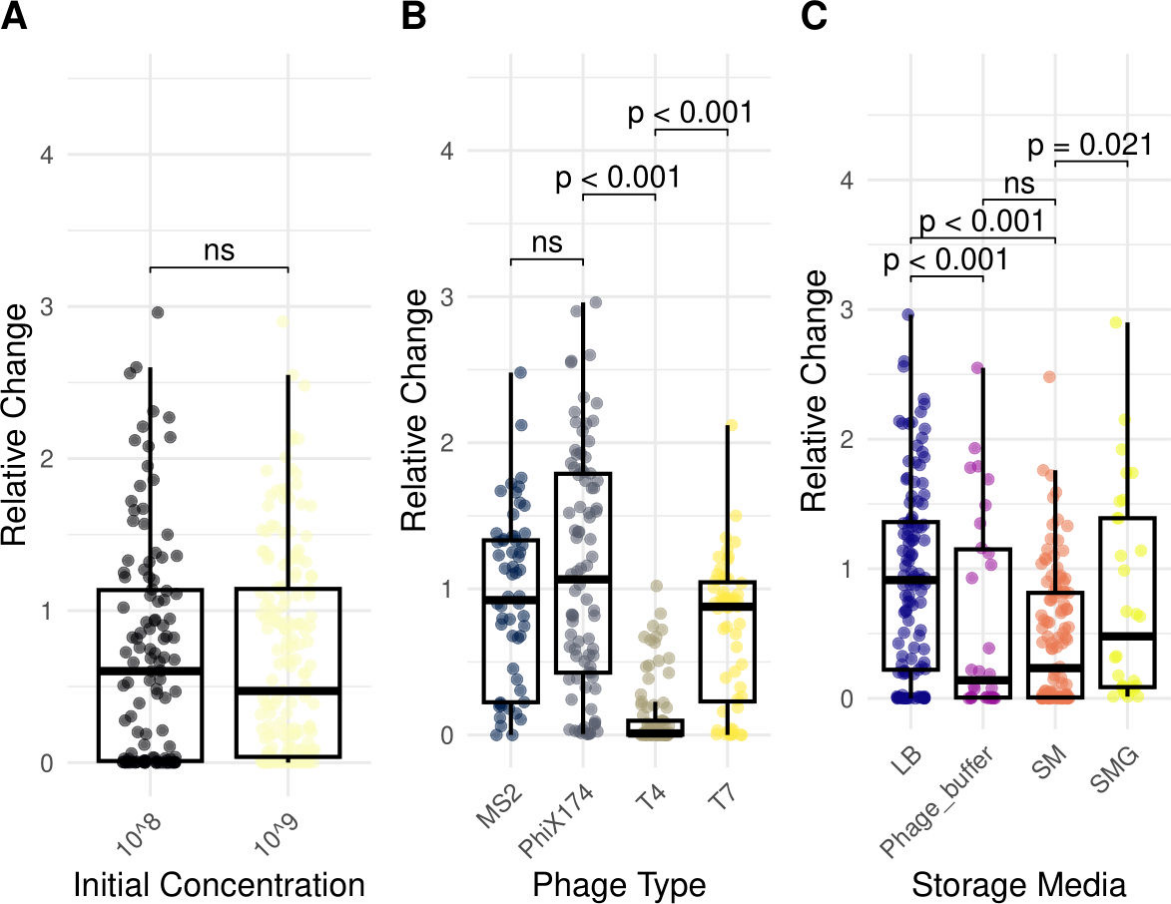
Comparison of relative phage titer change across variable categories. Box plots show the relative change in phage concentration, calculated as the final phage concentration at 24 months divided by the initial concentration. (**A**) Initial phage concentrations of 10^8^ PFU/mL (black) and 10^9^ PFU/mL (yellow). (**B**) Phage types: MS2 (dark blue), PhiX174 (black), T4 (gray), and T7 (yellow). (**C**) Storage solutions: LB (purple), phage buffer (pink), SM buffer (orange), and SMG (yellow). Each spot represents the mean relative change in phage concentration of three replicates. The horizontal lines in box plots denote median values. Statistical significance between groups was determined using Dunn’s test. *P*-values smaller than 0.05 were considered statistically significant.

Our analyses show that phage type and storage medium significantly impact the stability of phages under different storage conditions, while initial phage concentration does not have a significant effect (Fig. 1). T4 exhibited the lowest stability across storage conditions, whereas PhiX174 was less affected overall (Fig. 1B). When stored in different media, phages in LB showed the highest stability compared with those in phage buffer and SM (Fig. 1C). We then examined the effect of these conditions on each phage individually.

**Fig 2:**
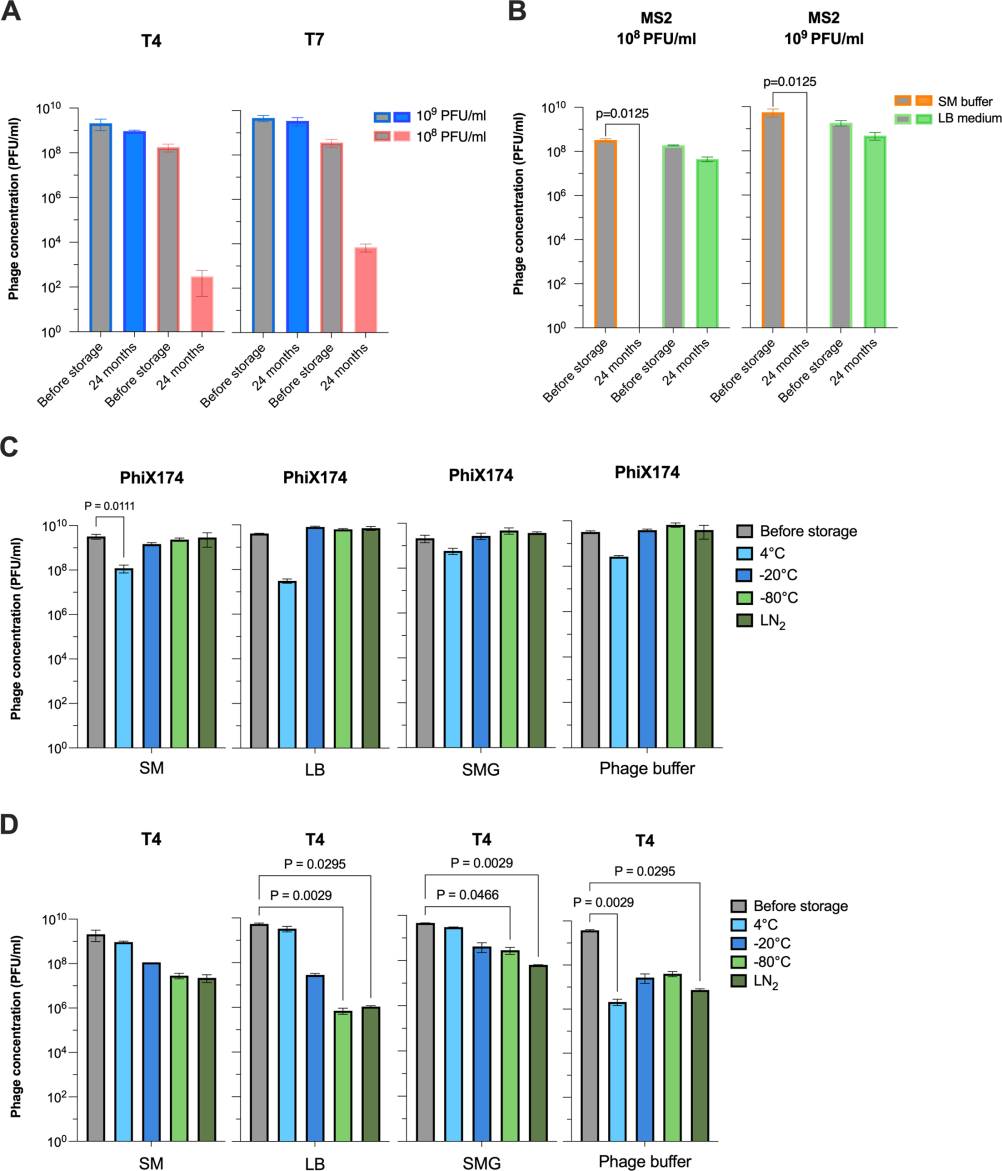
Effect of initial phage titer (**A**), storage buffer (**B**), and temperature (**C and D**) on phage stability. The bar graph illustrates the concentration of each phage measured before (gray) and after 24 months of storage. (**A**) Stability of T4 and T7 phages stored at 4°C in SM buffer. (**B**) Stability of MS2 phage stored at 4°C in different initial concentrations. (**C**) Stability of PhiX174 and (**D**) T4 phages at different storage temperatures. Phage concentration was quantified by plaque assay. Error bars represent the mean ± standard deviation of three technical replicates for each condition. Statistical significance was calculated using the Kruskal–Wallis test, followed by Dunn’s test; *P*-values smaller than 0.05 were considered statistically significant.

We observed that most phages remained stable at different initial concentrations under distinct storage conditions, except for T4 and T7 stored in SM buffer at 4°C. Their concentration decreased by 5.9 and 4.7 logs PFU/mL at the lower initial concentration (10^8^ PFU/mL), respectively, while at higher initial concentrations (10^9^ PFU/mL), the titer reduction was less than 0.5 log PFU/mL after 24 months (Fig. 2A). In addition, T4 stored in SM buffer at −20°C showed less stability at low initial concentration, with a decrease in titer 1.3 log PFU/mL higher compared with that of the high initial concentration (Table S2).

We then evaluated the effect of different storage solutions and found that MS2 stored in SM buffer at 4°C showed the lowest stability (Fig. 2B). There was a loss of up to 2.3 logs PFU/mL within the first week, and no active phage was detectable after 6 months of storage (Fig. S1B). However, the phage was relatively more stable when stored at a higher initial concentration, becoming undetectable only after 18 months. In addition, MS2 maintained its activity with a titer reduction of less than 1 log PFU/mL when stored in LB broth (Fig. 2B; Table S3). In contrast, T7 remained stable in most storage solutions, except when stored at −20°C or in low initial concentration (10^8^ PFU/mL) in SM buffer at 4°C, where its concentration in SM buffer was about 1 log PFU/mL lower than in LB after 24 months (Fig. 2A; Fig. S1). PhiX174 exhibited high stability in most solutions, with a decrease of less than 0.5 log PFU/mL when stored in solutions tested (Table S3). In contrast, T4 showed less stability at sub-zero temperatures, particularly in LB and phage buffer, where it experienced a reduction of up to 3.9 logs PFU/mL and around 2 logs PFU/mL, respectively. Interestingly, at 4°C, T4 remained stable in SM, SMG, and LB, exhibiting only a marginal decrease in titer of less than 0.5 log PFU/mL (Fig. S1A; Table S3).

We also studied the effect of lyophilization on the storage of MS2 and T4, which have shown lower stability in other conditions evaluated in the current study. They were freeze–dried in SM buffer with 0.5 M sucrose as a stabilizer and kept at 4°C. One day after lyophilization, T4’s titer decreased by 1 log PFU/mL, while MS2’s decreased by approximately 0.2 log PFU/mL (Fig. S2). The titer of both T4 and MS2 gradually declined by 2.9 and 3.3 logs PFU/mL, respectively, after 2 years of storage.

**Fig 3:**
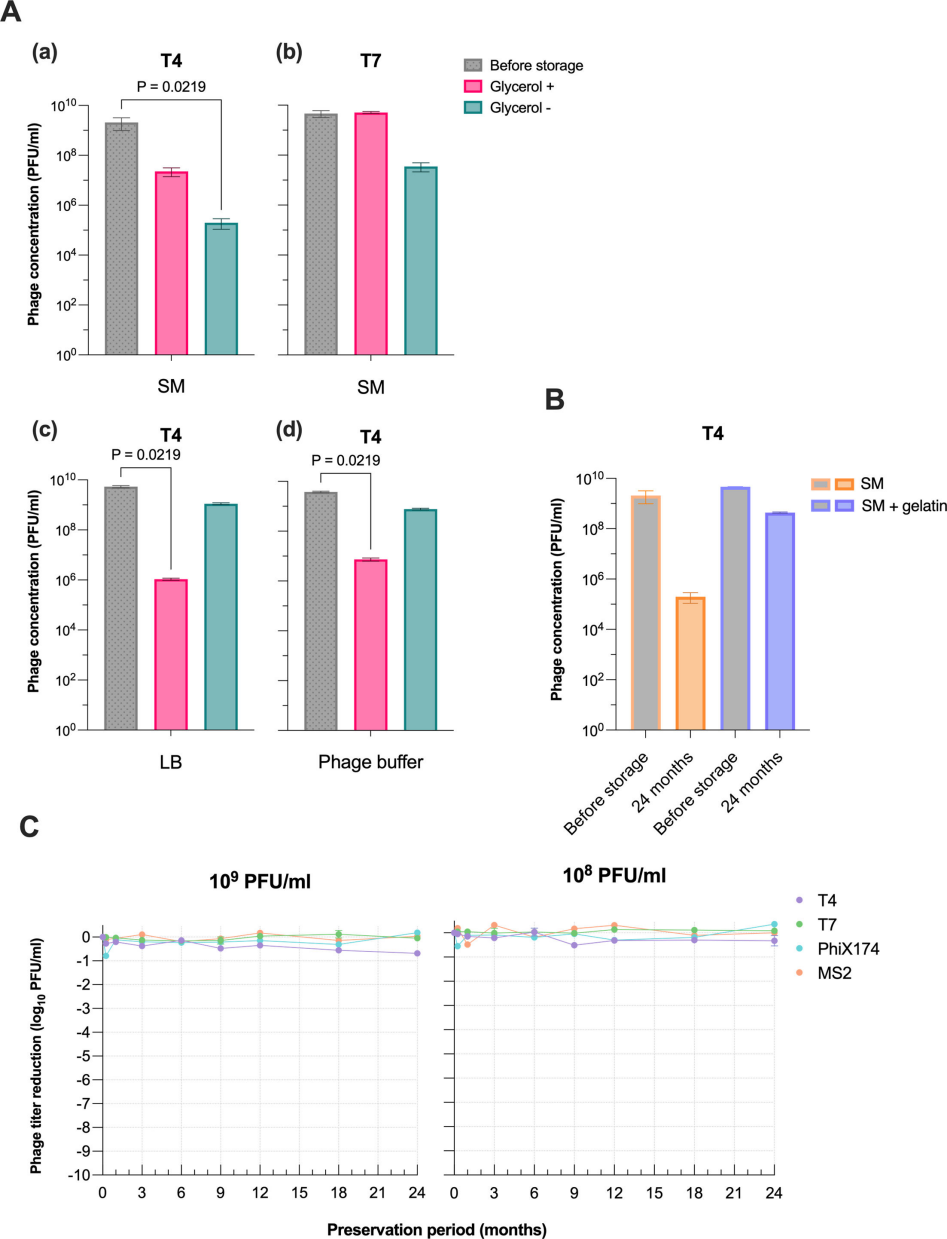
The impact of cryoprotectants on phage stability and the optimal storage condition for different phage types. The bar graph illustrates the concentration of each phage measured before and after 24 months of storage. (A) T4 and T7 in the initial 10^9^ PFU/mL concentration were snap frozen in LN_2_, then stored at −80°C. The storage solutions were SM buffer and LB broth with 20% glycerol (pink) and without glycerol (green). (**B)** T4 was stored in SM (orange) and SMG (purple) without glycerol at −80°C after snap freezing in LN_2_. (**C)** The line plots display the reduction in phage concentration (Log10 PFU/mL) over 24 months. Phages T4 (purple), T7 (green), PhiX174 (blue), and MS2 (orange) were snap frozen in LN_2_, followed by storage at −80°C. The initial concentrations were 10^9^ PFU/mL (left) and 10^8^ PFU/mL (right). Error bars represent the mean ± standard deviation of three replicates for each condition. Statistical significance was calculated using the Kruskal–Wallis test, followed by Dunn’s test; *P*-values smaller than 0.05 were considered statistically significant.

We found that both PhiX174 and T4 were the most sensitive phages to storage temperature. PhiX174 was more stable at sub-zero temperatures but experienced a 0.5–2 logs PFU/mL decrease at 4°C across diverse storage buffers (Fig. 2C). On the other hand, T4 showed high stability at 4°C but less stable at freezing temperatures, with its titer declining by 1 to 4 logs PFU/mL (Fig. 2D and Table S4). MS2 and T7 were relatively stable across most storage temperatures tested with minimal decrease in phage titer (Fig. S1A and B; Table S4), with the only exceptions mentioned above. We then assessed the effect of glycerol as a cryopreservative on the stability of phages, which were snap frozen in LN_2_. For the tailless phages MS2 and PhiX174, the presence or absence of glycerol generally resulted in titer reduction less than 0.5 log PFU/mL across most storage buffers (Table S5). However, in SM buffer, glycerol slightly improved MS2 stability, resulting in a 0.79 log PFU/mL higher titer. Similarly, T7 remained stable in LB regardless of the presence or absence of glycerol (Fig. S1A and B). Yet, when stored in SM buffer without glycerol for 24 months, it lost about 2 logs PFU/mL of its titer (Fig. 3A(b); Table S5). T4 displayed a similar result, with a significant decrease in titer when stored in SM buffer without glycerol (Fig. 3A(a)). Interestingly, the addition of glycerol in LB and phage buffer significantly impacted T4 stability, resulting in titer reductions of 3.7 and 2.7 logs PFU/mL, respectively (Fig. 3A(c,d); Table S5 ). Nonetheless, the titer of T4 stored in SM buffer without glycerol was 3 logs PFU/mL higher upon adding 0.01% gelatin (Fig. 3B). Both T4 and PhiX174 exhibited higher stability in SM buffer with supplemented gelatin (Fig. S1A; Table S5). Furthermore, we monitored the phage genome stability over the storage period using qPCR. Despite the qPCR results showing high variability at different time points, the phage genome quantification did not reveal a trend toward degradation (Fig. S3).

## DISCUSSION

There is a growing interest in using phage therapy as a solution to the global problem of antimicrobial resistance (1). To provide phage therapy in a timely manner, it is helpful to create a phage collection for the long-term preservation of isolated and characterized phages. However, preserving phages for the long term can be challenging due to their high diversity and lack of standard storage methods. Here, we stored four phages representing different phage morphotypes (Myovirus, Podovirus, Levivirus, and Microvirus) under distinct conditions for 2 years (Table S6). We then assessed the effect of these conditions on the stability of the phages over time.

Our study indicates that the choice of storage buffer significantly affects phage stability, with phages showing higher titer loss in SM buffer compared with LB broth. The high salt and protein concentration in LB may help stabilize phage particles. We found that tailed phages generally exhibit higher stability when stored in chemical-based buffers at higher titers and 4°C. T4, a Myovirus, was the most sensitive to freezing temperatures among the phages studied. The long contractile tail of Myoviruses may contract at sub-zero temperatures, leading to a loss of phage infectivity (12, 23, 24). In addition, the osmotic stress during freezing may cause the tail sheaths to detach in T4 (25, 26). The length of the phages‘ tails is also linked to their susceptibility to freezing, with Myo- and Siphoviruses being more prone to tail damage compared with Podoviruses against mechanical stresses (27). Siphoviruses, the most abundant tailed phages, were excluded from this study but are known to exhibit high stability under various storage conditions (26, 28, 29).

We also evaluated the impact of cryoprotectants, such as glycerol and gelatin, on phage stability. Interestingly, although glycerol is widely used as a standard practice for the cryopreservation of biological samples, our results showed that it negatively affects T4 in LB and phage buffer, likely due to altered osmotic pressure and ionic strength resulting from the interaction between chemical compositions of the solutions with glycerol (30, 31). This is consistent with previous findings that also reported a detrimental impact of glycerol on phage stability at −80°C and in liquid nitrogen (5). Gelatin, in contrast, enhanced phage stability, most probably due to its function as a protective matrix during freezing. We have also explored the possibility of using lyophilization to preserve phages for the long term. While this method is commonly used in the industry, it is less frequently used in research laboratories because of the limited availability of freeze-dryers. Our findings show that lyophilized phages lost their viability, likely due to suboptimal storage humidity. This resulted in high moisture content, which had a negative impact on phage activity (32, 33).

Overall, we found that snap freezing in liquid nitrogen followed by storage at −80°C using LB as the storage buffer without glycerol effectively maintained the viability of all four phages among the 30 storage conditions evaluated. In addition, we observed that the titer reduction mainly occurred within the first month after storage, followed by a gradual decline. This highlights the importance of early measurements of phage concentrations during storage to predict the long-term stability of phages, particularly tailed phages. The storage solutions evaluated in this study are tailored for laboratory applications and phage banks. However, for therapeutic applications, these buffers must be replaced with formulations approved for clinical use, ensuring compliance with regulatory standards and addressing requirements, such as endotoxin reduction (34). In addition, future research is necessary to expand on our findings by examining a wider diversity of phages. This will provide a deeper understanding of how genomic and structural diversity influences phage stability, particularly in clinical-grade preparations.

## SUPPLEMENTAL MATERIAL

Supplemental figures spectrum.01442-24-s0001.pdf Fig. S1 to S3.

Supplemental tables spectrum.01442-24-s0002.xlsx Tables S1 to S6.

ASM does not own the copyrights to Supplemental Material that may be linked to, or accessed through, an article. The authors have granted ASM a non-exclusive, world-wide license to publish the Supplemental Material files. Please contact the corresponding author directly for reuse.
