# Supplementary material for: Comparative evaluation of long-term preservation methods for morphologically distinct bacteriophages
Source: Microbiol Spectr. 2025 Apr 16;13(7):e01442-24. doi: 10.1128/spectrum.01442-24 (PMC12211016; doi:10.1128/spectrum.01442-24)
Supplement: Supplemental figures — Fig. S1 to S3. [file spectrum.01442-24-s0001.pdf]

Supplemental Figures

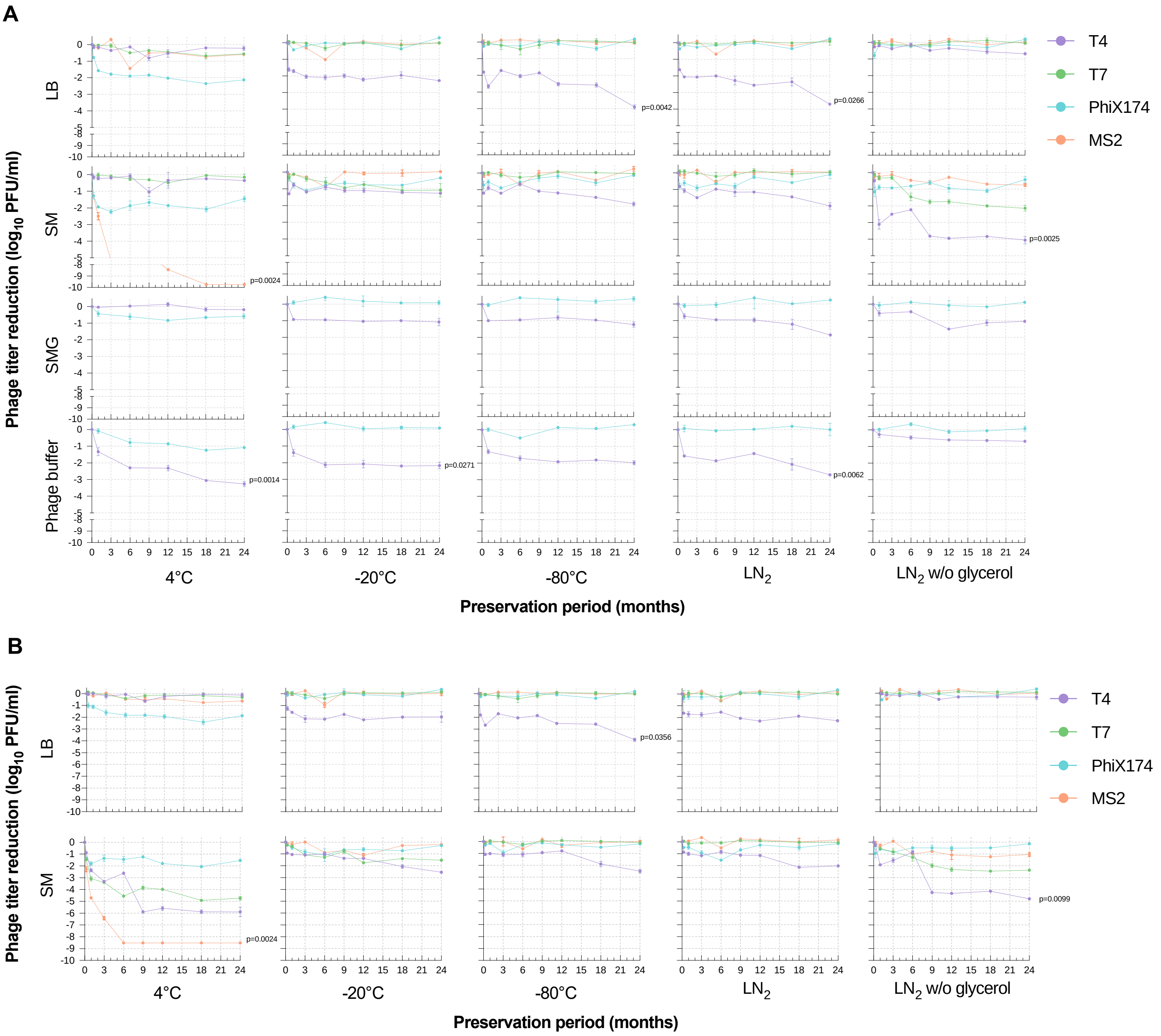

Figure S1. Stability of four phages under various preservation temperatures and storage buffers. The line plots display the reduction in phage concentration (Log_{10} PFU/mL) over 24 months of storage under five different preservation conditions: 4°C, -20°C, -80°C, LN_2, and LN_2 without glycerol, across four different storage buffers: LB (top row), SM (second row), SMG (third row), and phage buffer (fourth row). Data are shown for two initial stock concentrations: (A) 10^8 PFU/mL and (B) 10^9 PFU/mL. Each panel represents a unique combination of storage buffer and temperature. Four phages are presented by different colored lines: T4 (purple), T7 (green), PhiX174 (blue), and MS2 (orange). The x-axis shows the preservation period in months, and the y-axis shows the log reduction in phage concentration, quantified using plaque assay. Error bars represent the mean ± standard deviation of three replicates for each condition. Statistical significance was calculated using the Kruskal-Wallis test, followed by Dunn's test to compare phage concentrations at month 24 and before preservation; *P*-values smaller than 0.05 were considered statistically significant.

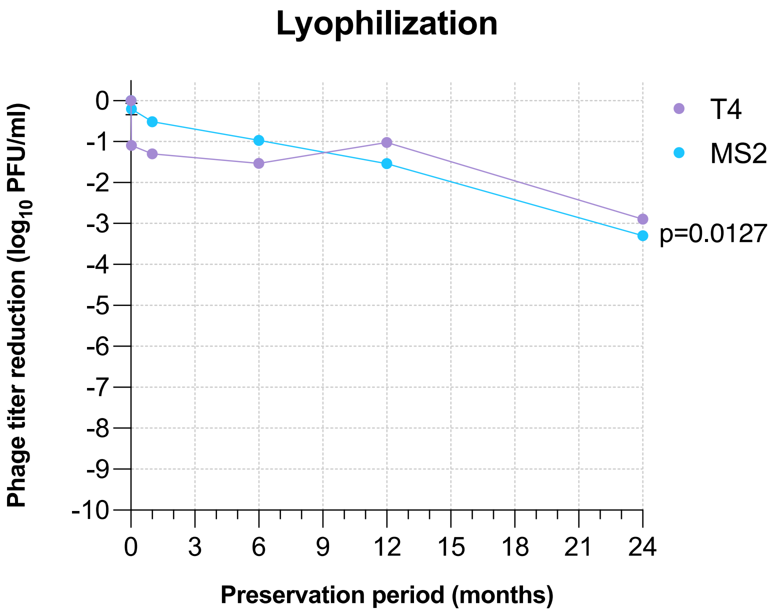

Figure S2. Phage stability of T4 and MS2 after lyophilization. The line plot depicts the reduction in phage concentration of phage T4 and MS2 after lyophilization in SM buffer supplemented with 0.5 M sucrose, followed by storing at 4°C. The initial concentration was 10^{10} PFU/mL. T4 and MS2 phages are presented by purple and blue lines, respectively. The x-axis shows the preservation period in months, and the y-axis shows the log reduction in phage concentration, quantified using plaque assay. Error bars represent the mean ± standard deviation of three replicates for each condition. Statistical significance was calculated using the Kruskal-Wallis test, followed by Dunn's test to compare phage concentrations at month 24 and before preservation; *P*-values smaller than 0.05 were considered statistically significant.

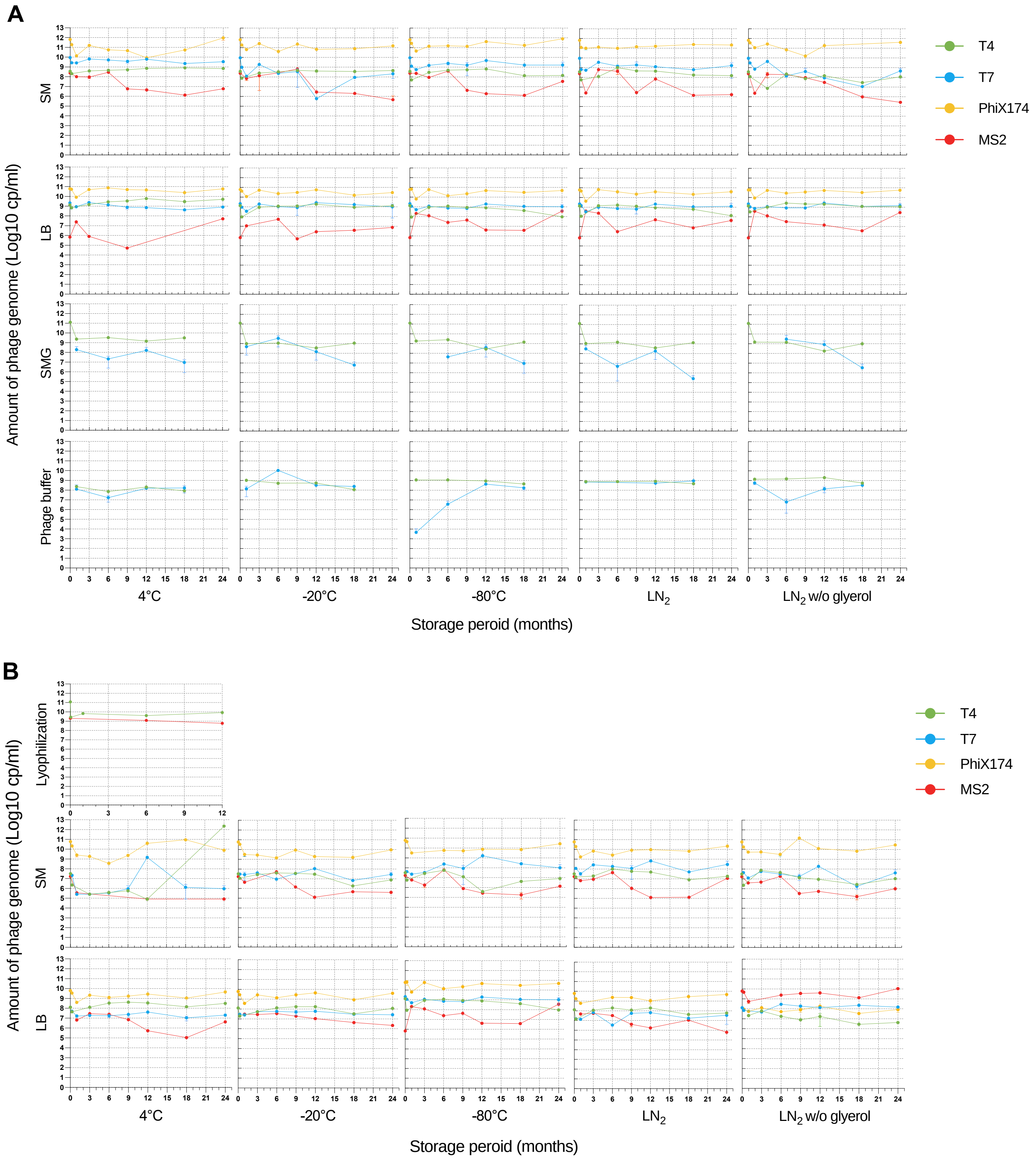

Figure S3. Amount of phage genome in stocks quantified by qPCR during the 24 months of preservation. The phage genome stability was represented by the residual amount of phage genome of T4 (green), T7 (blue), PHiX174 (yellow) and MS2 (red) quantified with qPCR. The ct values were converted to Log10 molecular copies per mL (Log10 cp/mL) of the stocks. The initial concentrations of the samples were (A) 10^9 PFU/mL and (B) 10^8 PFU/mL. The samples were treated with DNase and proteinase K, followed by DNA and RNA extraction. Error bars represent the standard deviation of two technical replicates.
